# *Legal Positivism’s Internal Morality* ^†^

**DOI:** 10.1093/ojls/gqac030

**Published:** 2022-12-21

**Authors:** Javier Gallego

**Affiliations:** University College, Oxford; University Adolfo Ibáñez, Chile

**Keywords:** legal positivism, legality, rule of law, unjust law, legal theory

## Abstract

This article examines the jurisprudential arguments elaborated in David Dyzenhaus’s *The Long Arc of Legality*. In particular, it looks into the main claim of the book: that the fact of ‘very unjust laws’ is central to illuminating the idea of law’s authority, the elaboration of which Dyzenhaus takes to be the purpose of legal theory. The article analyses Dyzenhaus’s own normative proposal in this matter, which consists of a version of legal positivism committed to Lon Fuller’s principles of the internal morality of law, with the corollary of a conception of the judicial role as bound to a duty to apply these internal principles of legality when exercising their main function. While I cast some doubts on the feasibility of constructing the judge’s function that way, in the end I celebrate Dyzenhaus’s attempt at refining legal positivism’s identity, especially in light of the ongoing debate with contemporary anti-positivism.

the puzzle of very unjust law is

a permanent problem for legal theory.

One might even say with only slight

exaggeration that it is *the* problem for legal theory…

David Dyzenhaus^1^

## 1. Introduction

The bold statement in the epigraph gives the impression that the idea of a ‘very unjust law’ is the subject matter of Dyzenhaus’s ambitious book. But the fact is that ‘very unjust law’—that is, a law that is deeply immoral—is only instrumental to delineating the concept of legal authority, which is the ultimate aim of *The Long Arc of Legality*.

Indeed, Dyzenhaus adds that the problem of ‘very unjust law’ is—or can be considered—*the* problem of legal theory (his emphasis), as long as we understand legal theory (or the philosophy of law) to be ‘principally about explaining law’s authority’.^2^ And elsewhere he says that ‘the problem of unjust law … serves mainly to point to a deeper problem about how to reconcile our intuitions that law is both a matter of fact and a matter of authority’.^3^

Before addressing the question of how the fact of a ‘very unjust law’ illuminates the explanation of law’s authority, we should first reflect on what Dyzenhaus claims legal theory is about.

A ‘legal theory’, or a ‘theory of law’, usually addresses a number of philosophical questions: there is the issue of legal ontology—what makes some institutional action count as ‘law’; the issue of legal normativity—how institutional action deemed ‘legal’ changes the normative situation of agents; and the issue of legal content—what part of such institutional action should everyone treat as legal statements, and how these statements are to be extracted from institutional materials.^4^ Dyzenhaus does not clarify which of these questions the book aims to address. The declared purpose of the book from the start is to solve the deadlock between legal positivism and natural law regarding ‘the puzzle of law’s authority’, that is, the claim that ‘law is both a matter of right and might’.^5^ Early on, he invokes Hart’s famous phrase that ‘Law surely is more than the gunman situation writ large’^6^ as implying that Hart’s theory of law contains a theory of legitimate political authority. The intuition appears to be that law cannot simply consist of institutional directives backed by coercion (‘might’), but must make a moral claim to rule subjects in a proper manner (‘right’).

Methodologically speaking, the book tells a story through the voice of three legal positivist: Hobbes, Kelsen and Hart. In Dyzenhaus’s opinion, these three philosophers all have some contribution to make to a view where law is presented as something more than just ‘might’ and including ‘right’. From this choice of authors, the message is clear: Dyzenhaus believes that legal positivism is the better positioned to explain law as a system of ruling with a morally valuable purpose (to rule in a proper manner). Thus, it would make sense to ascribe to Dyzenhaus some of the main tenets of legal positivism, at least with regard to legal ontology and legal content. This entails primarily two theses: (i) that the existence of law depends on some specific institutional actions with communicative content, which by convention or some other social fact are understood by a community to produce properly legal facts; and (ii) that the content of the law correlates with the textual content of such communicative acts. Furthermore, given that for Dyzenhaus to uncover law’s authority implies reconstructing law as more than just ‘might’, it is safe to impute him a third thesis, which is not unique to legal positivism: (iii) that law is inherently coercive.

Curiously, the legal positivist that famously built a conception of law around the concept of authority, Joseph Raz, does not figure in Dyzenhaus’s pantheon. Quite the contrary, the book argues that Raz’s idea of legal authority is misconceived. Raz meant to complement the reductionist explanation of legality of legal positivists like Austin and Hart—who ‘reduce the grounds of legality to social facts’^7^—with a theory of the normative difference that institutional action deemed ‘legal’ is supposed to make in the subjects of law: law is supposed ‘to mediate between people and the reasons that apply to them’.^8^ In that way, Raz made the position of the ‘subject of law’ a central element of a theory of law: law is not just brute coercion, it necessarily speaks to subjects’ reasons.^9^ Dyzenhaus follows that path in some sense when he claims that law’s authority ‘is a matter both of what legal subjects in fact accept and what they have reason to accept’.^10^

Broadly speaking, then, both Raz and Dyzenhaus believe that legal theory should reconstruct law as institutional action morally justifiable to each person. But at the same time, Dyzenhaus believes that Raz did not push this idea of justifiability far enough. The idea that law’s authority is somehow demonstrated by the fact that the law contributes to changing the normative landscape by providing normative reasons to its subjects is insufficient. Law’s legitimate authority is not assured by the law simply claiming it, the law must actually *have it*.^11^ When can we say, then, that the law actually ‘has’ legitimate authority?

In Dyzenhaus’s view, to actually have it, the law must assert its authority in contexts of application: it is crucial that officials in charge of applying the law somehow assert the authority of law by making law’s demands morally justifiable to each person. This means that the question of law’s authority (whether the law actually has it) appears in front of a law-applier every time she has to apply a law to a particular case. Besides considering facts and applicable legal materials, the law-applier must somehow be able to respond to a question that is posed from the standpoint of the legal subject: ‘But how can that be law for me?’^12^ This suggests that, for a law-applier to apply a law to a case, she must consider whether the law in question is really one the subject has reason to accept. The valid and applicable law, then, must be subject to an evaluative standard.

Dyzenhaus draws this standard for evaluating institutional action from Lon Fuller’s principles of legality or rule of law—a set of normative demands imposed, at the level of theory, into the formal structure of the laws of a given legal order: that laws ought to be general, public, prospective, obeyable, coherent and so on.^13^ Dyzenhaus even claims that the whole of his book can be understood as an attempt to make sense of the idea that such normative standards define ‘legality’,^14^ that is, define what ‘law’ is. This takes place as a process of transformation. In Dyzenhaus’s words, ‘legality mediates the conversion of public policy into the enacted law’, and by this mediation ‘political judgments are converted into legal content’.^15^ Carrying out this mediation is the law-applier’s role. But note that this is so because the principles of legality are understood to be constitutive of law’s claim to rule in a morally justified manner. So, we can see this as a modified version of Raz’s idea that law claims authority to rule. The law claims this authority not (or not only) because it is better positioned to solve coordination problems (which is, in Raz’s view, what ultimately explains the law’s normative contribution), but rather because it claims to be ruling through legislation that is general, public, prospective and so on. Since we cannot trust legislators on this, the law-applier must have the ultimate say on the matter.^16^

The project is ambitious not only in terms of the amount of philosophical work it aims to cover, considering it aims to extract this idea of legal authority from the work of Hobbes, Kelsen and Hart. It is also ambitious in the breadth of substantive elements the theory is supposed to have an impact on. The main goal of the book is to establish the grounds for a normative theory of the law-applier’s role when applying law, but the view has corollaries for a theory of legal normativity (of the law’s contribution to subjects’ practical reasons),^17^ and also goes well beyond legislated law. It expands to the constitutional order^18^ and the international legal order,^19^ both of which play a major role in the theory. Both are fundamental for a complete account of law’s authority in Dyzenhaus’s understanding of the idea. This, of course, reflects decades of work devoted to such issues, which Dyzenhaus now wants to bring together in book format.

In what follows, I will not say much (nothing really) about the contribution the book expects to make in these fields, but will concentrate instead on the basic jurisprudential claims it makes. The second section introduces the idea of a ‘very unjust law’, which poses a sort of litmus test for the affirmation of law’s authority in contexts of application. I rely on a fairly intuitive understanding of what makes a law ‘very unjust’, for the section’s aim is to introduce the authors and ideas Dyzenhaus relies on in his book to confront the problem he presents. The third section revises Dyzenhaus’s position towards legal positivism, which is marked by a dramatical shift, from frontal opposition to legal positivism in his early work to a gradual embracement in his later work. This analysis sheds light on the reasons that led Dyzenhaus to the position he puts forward in this book. The fourth section analyses the normative proposal of the book: that judges ought to apply the principles of legality to ascertain law’s authority. The fifth section dives into some of the claims Dyzenhaus makes about contemporary anti-positivism. The sixth section offers some conclusions.

## 2. Very Unjust Law

Why is ‘very unjust law’ so central to jurisprudence? Recall that, in Dyzenhaus’s view, legal theory is about explaining law’s legitimate authority, that is, explaining how institutional force is transformed into legal governance. A ‘very unjust law’ appears to pose a problem for that task since it presents a case where a valid and applicable law is so deeply immoral that it appears not to be justifiable as an exercise of legitimate authority.^20^ This opens two possibilities: either one declares the law not to be ‘really law’ because it fails some evaluative standard, or one declares it is a law but not one a judge^21^ should enforce or a citizen should obey.

Early in the book, Dyzenhaus makes the claim that neither legal positivism nor natural law theory, canonically understood, is able to properly address the problem that very unjust laws pose. Thus, very unjust laws present a ‘puzzle’ for legal theory.^22^ Of course, positivists can say that very unjust laws are valid laws but not laws that a judge should apply. Natural lawyers can say they are not laws (which has the same practical consequence: judges ought not to apply them). But Dyzenhaus thinks both reactions are missing something important, albeit for different reasons. Legal positivism seems to contradict itself by reacting this way to a very unjust law, since positivists are supposed to be committed to the separation of law and morality. Pinning down what is wrong with the natural lawyer’s reaction is more difficult, because here Dyzenhaus’s own ideological biases contaminate the evaluation. Although the natural lawyer’s reaction to the fact of very unjust laws might be coherent overall, Dyzenhaus disavows it because the natural lawyer gives up positive law to define legality solely through morality.

So, the ‘puzzle’ is only a real problem for legal positivists, since natural lawyers have a straightforward answer to the ‘puzzle’. Different versions of the ‘natural law thesis’ treat unjust laws either as non-laws (as in the *lex iniusta non est lex* maxim) or as defective cases of law.^23^ Now, as I said, given that Dyzenhaus draws elements from Hobbes, Kelsen and Hart to construct his approach, it is safe to assume that the goal of the book, more than solving an apparent deadlock between two legal theories, is actually to reconstruct legal positivism so that it can coherently respond to the so-called puzzle of very unjust law.

That the purpose of the book is to reconstruct legal positivism in a better light is not only evident from the choice of authors to tell the story. It also shows in the fact that, early in the book, Dyzenhaus dismisses Fuller and Dworkin jointly as exponents of a natural law tradition that necessarily links law to morality,^24^ and is thus incapable of reconstructing the law in a way that solves the puzzle. But the matter is not so simple. Fuller and Dworkin are actually two major characters in Dyzenhaus’s narrative, and the book refers to them and engages with their work to almost the same degree as it does with Hobbes, Kelsen and Hart. While Dyzenhaus gets from Hobbes the idea that the legitimacy of the legal order sprouts from a social contract between the ruler and the subjects—one which demands from the ruler that it sustains and advances the interests of the subjects^25^—and gets from Kelsen an overall pragmatic approach to legal theorising,^26^ the substantive part of the argument relies extensively on Fuller and Dworkin jointly.

At this point, it would be useful to present Dyzenhaus’s core jurisprudential argument in summarised terms. The basic idea is that legal positivism, in its best version, is not only committed to a legal ontology reducible to social facts, but also to the idea that a properly legal order depends on the legal system complying with something like Fuller’s principles of the rule of law. Dyzenhaus’s next move is to construct a normative theory of the judicial function that makes the application of those principles of legality a central component of such function.^27^ For this purpose, he uses a Dworkinian framework to ascertain the judge’s duty to go beyond enacted law and look into these principles of legality when exercising their function.

This makes one wonder: why are Fuller and Dworkin not in the ‘long arc of legality’? Perhaps having all these names together was too long for a book subtitle, but of course the main reason is ideological and lies in Dyzenhaus’s commitment to legal positivism’s core tenets. Now, as I hinted already, in Dyzenhaus’s understanding, legal positivism presents law as having a moral purpose: to rule subjects properly, through laws that are public, prospective and so on. Invoking Fuller’s notion, we would say that law has an ‘internal morality’ (evidenced in its commitment to the morally valuable aim of ruling subjects following the principles of legality), and legal positivism, in its best version, is committed to this internal morality. Fuller and Dworkin aid Dyzenhaus in constructing this interpretation of legal positivism, but their place in the tradition itself is a matter of controversy, which might explain why they are not in ‘the long arc of legality’.

This also probably explains Hart’s place in ‘the long arc of legality’. While Dyzenhaus could have placed Fuller alongside Hobbes and Kelsen in his narrative, he chooses instead to vindicate some often-neglected passages from Hart’s *The Concept of Law*,^28^ especially its chapter 9, where Hart tentatively puts forward a Fullerian idea in a shorter version: that for law to serve justice, it must provide for rules that are general, public, intelligible, prospective and obeyable by subjects.^29^

Now, before determining whether Dyzenhaus succeeds in bringing the elements of ‘legal positivism’s internal morality’ to his normative theory of the judicial function, we should take a historical detour into his shifting position towards legal positivism. Indeed, Dyzenhaus has shifted his position dramatically towards legal positivism over the years, going from a stance of frontal opposition in his early work^30^ to a gradual embracement in his later work. Two reasons explain this shift: first, his position on Dworkin’s jurisprudence *vis-à-vis* legal positivism, and secondly, the fact that in his early work his concern was with unjust *legal systems*, drifting into a concern with unjust *laws* in his later work. This detour will help explain, I hope, the reasons behind his current ideological commitment to legal positivism and, further, whether his ultimate project succeeds or not.

## 3. Dyzenhaus on Legal Positivism (and Dworkin)

In his early systematic elaboration of the problem of very unjust law,^31^ Dyzenhaus accused the legal positivism of Hobbesian roots of authoritarian tendencies,^32^ because of its commitment to the idea that law should be applied by judges and obeyed by subjects simply as the commands of a commander. When analysing Hart and Raz’s positivism, he reached the conclusion that, although their theories of legality offered an alternative to the commander’s will as the source of legality, in the end neither could escape the authoritarian tendencies of the ‘Hobbesian insistence on the preemptive quality of law’.^33^ This is not because of their views on legal validity, but because of the consequence of legal positivism’s theory of legality on the question of the normativity of the judicial function. The legal positivist’s response to the question about what judges are supposed to do when faced with a very unjust law—a ‘hard case’—is simply ‘exercise discretion’. This is what Dyzenhaus (following Dworkin) called a ‘plain fact approach’ to the judicial function. He then concluded that,

contemporary positivists [Hart and Raz] have not distanced themselves far enough from the command theory to escape the charge that their view of law encourages the plain fact approach. And they compound this by failing to offer a doctrine of judicial responsibility which could give judges an acceptable *legal* reason to exclude the kinds of plain fact which in a wicked legal system make the system into the worst system it could be …

We can now see that the anti-authoritarian stance contemporary legal positivism adopts stands in a pragmatic tension with the positivist conception of law … [W]hile contemporary positivists want judges and citizens not to be authoritarian, they offer a conception of law which as a matter of practice will be implemented by the judges of a wicked legal system in an authoritarian way.^34^

Back then, Dyzenhaus’s preferred alternative was Dworkin’s theory, which he understood as ‘constructive and practice-oriented’,^35^ and thus not constrained by positive law, and for this reason better suited to provide a normative theory of the judicial function in the face of very unjust law.

But over the years, Dyzenhaus’s position changed. While he became sceptical of Dworkin’s resources to confront the phenomenon of very unjust law, at the same time he began to acknowledge that perhaps it was legal positivism (in Hart’s version at least) that was the better suited to confront very unjust law.

Julie Dickson also saw this. She has said that it is ‘somewhat ironic’ that legal philosophers like Finnis and Dworkin, who argue

that in order properly to identify and understand law, we must take a stance on, and understand it in terms of, its morally valuable purpose or point, claim not to be terribly interested in the question of whether purported instances of law which fail to achieve that purpose … are indeed still instances of law.

Dickson also noted that, in contrast, it was Hart who ‘thought it of vital importance that we face head-on the issue of whether the moral iniquity of rules of law renders them any less legal’.^36^

Dickson believes this is so because legal positivists in general adopt a methodology for legal theorising she calls ‘Indirectly Evaluative’, which is committed to a two-stage inquiry when attempting to define law. The first stage involves identifying the necessary and important features of any purported legal system without engaging in moral evaluation of those features. Settling this first issue allows the theorist to engage in properly moral evaluation in the second stage.^37^ It is crucial that in the first stage of inquiry the legal theorist takes into account an important duality: law’s social existence and law’s normative nature. For Dickson, it is of the essence of legal theorisation that the theorist keeps the balance between law’s normative claims and law’s real existence in society as a set of institutions.^38^ By conditioning the identification of ‘law’ proper to the verification of law’s normativity, Dickson believes, a legal theory fails to keep the balance and surrenders legal theorisation to moral evaluation. This she sees is the case for Finnis, Dworkin and contemporary anti-positivists who elaborate Dworkin’s interpretivism.^39^

But the important point for our purposes is that, for Dickson, Indirectly Evaluative Legal Theory is what allows the theorist to keep a healthy distance—‘an attitude of due wariness’^40^—to morally problematic laws, thus facilitating moral evaluation and moral criticism of laws.^41^ Dickson exemplifies with the Jim Crow laws in the United States, the legal directives constituting and supporting apartheid in South Africa, and laws criminalising homosexuality.^42^ These examples should appeal to Dyzenhaus, and some of them appear in his book. But my hypothesis here is that Dyzenhaus must have come to the same realisation, that only legal positivism provides the necessary attitude of due wariness to what he calls ‘very unjust laws’, and that this explains him losing faith in Dworkin’s theory as the solution for the problem of unjust law.^43^

But there is more to this. We should consider that while Dyzenhaus was changing his views about legal positivism, Dworkin’s theory was also evolving. In *Justice for Hedgehogs*,^44^ Dworkin famously advocated for the one-system view of law and morality. Dyzenhaus believes this move is what renders Dworkin’s theory ultimately unable to coherently react to very unjust law. For Dyzenhaus, very unjust law presents a ‘puzzle’ for Dworkin because he has to choose between saying that the very unjust law is law ‘despite the fact that’ the law does not supply its subjects with moral reasons or saying that it is not law *precisely because* it fails to give these subjects moral reasons. But why would it be a problem for Dworkin to say that a very unjust law is not law for its subjects given that it fails to give them moral reasons? Dyzenhaus believes it is so because by saying this Dworkin ‘consigns the problem of the injustice of unjust law to morality, thus presupposing the two-system picture of the relationship between law and morality that he wished to reject’.^45^

Dickson would have an alternative explanation to the ‘puzzle’ that affects Dworkin’s theory. It is simply that he—like Finnis—is engaging in (what Dickson calls) ‘Directly Evaluative Legal Theory’, which subordinates the question of law’s ontology (with consequences for the theory’s take on the question of the normativity of the judicial function of applying the law) to the normative evaluation of the moral goodness of laws, thus leaving no real space for a critical assessment of the morality of laws.

Recall that, for Dyzenhaus, it is Dworkin’s adoption of the ‘one-system view’ that ultimately renders his theory unable to escape the ‘puzzle’. This is important because, as we saw, Dyzenhaus once thought that it was Dworkin’s ‘constructive and practice-oriented’ theory of law (and of adjudication) that made it a better candidate than legal positivism to face the problem of very unjust law. And even when Dworkin’s ‘one-system view’ now makes the ‘puzzle’ inescapable, Dyzenhaus still thinks he can save Dworkin from himself by adopting a Dworkinian theory of adjudication that, instead of moral rights, introduces Fuller’s principles of legality as the background principles the judge must look for to assess the legality of putative laws.^46^

Dyzenhaus’s somewhat ambivalent position towards Dworkin is explained by the fact that he has always read Dworkin’s theory through a positivist lens. This is an extended practice among legal positivists, who understand Dworkin’s theory as a theory of legal interpretation instead of a metaphysical theory about legal content, as contemporary anti-positivists do. On the legal positivist reading, the idea of ‘integrity’ that Dworkin elaborates in *Law’s Empire*^47^ entails a demand addressed to law-appliers to engage in a constructive interpretation of the law, taking into account some background moral or legal principles that aid interpreters when some correction is needed in the process of applying posited law.^48^ Legal positivists in general claim to obtain this idea from Dworkin’s essay ‘Model of Rules I’,^49^ and this understanding of principles, as materials standing alongside legal rules, pervades their reading of Dworkin’s later work. Contemporary anti-positivists, by contrast, pay far more attention to Dworkin’s ‘Model of Rules II’,^50^ which contains Dworkin’s attack on the idea that legal normativity flows from something like Hart’s practice theory, arguing instead that legal normativity is not different from the normativity of moral norms. Anti-positivists draw some conclusions for the determination of legal content from that. Dworkin-II legal theorists, let us call them, understand principles to be the sole source of legal normativity (and of legal content). For them, principles are prior, not correctives of the legal interpretation of posited law.^51^

Dyzenhaus is a Dworkin-I type of Dworkinian. For him, Dworkin’s theory amounts to a pragmatic theory of adjudication, whereby posited law is prior, and principles come to the rescue later in the legal interpretative process. This explains why he cannot quite conciliate Dworkin’s move to the ‘one-system view’, which, of course, Dworkin-II-type Dworkinians have no problem embracing, since for them it is the most coherent final statement of what Dworkin has been saying since ‘The Model of Rules II’ (and, in fact, since ‘The Model of Rules I’). We will return to Dworkin-II legal theorists in a moment.

## 4. The Judicial Function

I said that Dyzenhaus believes Dworkin can be rescued from himself by preserving his framework of judicial interpretation but replacing moral principles with Fuller’s principles of legality. I also hinted before at the fact that there is a major shift in the subject matter of Dyzenhaus’s inquiry. While, in his early work, he was concerned with wicked or unjust *legal systems* (see the long quote in the previous section), in his recent work, he has shifted to a concern with unjust *laws*. Since the concern is now individual unjust laws, by definition the argument must presuppose a fairly healthy legal system.

This changes things. If we determine, by whatever means commonly accepted, that a given legal system is widely wicked or unjust, then our response to the question of a judge’s responsibilities in such a system will be somewhat biased, given that we would have already established that the system as a whole is wicked or unjust. This is what probably attracted Dyzenhaus to Dworkin in the first place: the possibility of going beyond the posited laws of a widely wicked legal system and looking instead for principles to exercise the judicial function. But now that the concern is with unjust *laws* within a healthy *legal system*, that escape route appears to make less sense.

So, Dyzenhaus preserves from Dworkin only a method of legal reasoning. The method is tasked with the goal of evaluating whether individual laws ascertain a legal system’s claim to rule in accordance with its internal morality, and this is where Fuller’s idea of the rule of law comes in. But there is a problem. Fuller’s principles of the internal morality of law were not meant to be invoked by law-appliers deciding cases. Fuller did develop a theory of legal interpretation far opposed to what Dyzenhaus would call a plain fact view of adjudication, emphasising a purposive approach to legal materials, and stating, among other things, that the role of the judge is ‘helping to create a body of common morality’,^52^ and that the application of statutory law must be done ‘in accordance with principles of interpretation that are appropriate to [the legal officials’] position in the whole legal order’.^53^ But, as far as I can see, Fuller never explicitly linked these views on adjudication with his theory of the rule of law. The subject matter of Fuller’s proposed normative evaluation was always a legal system.

This fact has made it easier for some legal positivists to embrace Fuller’s idea of the internal morality of law, even as a sort of litmus test for the validity of laws. Kristen Rundle explains it thus:

Fuller’s gesture might be read as offering a standard for legal validity that is grounded in a positivist, source-based test, and which accepts this test as appropriate to the majority of cases, but which then insists that the formal health of the putative law … should be the arbiter of how far that acceptance ought to extend. In short, we can accept as valid law that which a source-based factual test for legal validity pronounces to be such, until and unless a point is reached when *the legal order* from which that law emanates is so plagued by formal pathologies that its output … should be denied that status.^54^

Legal positivists who embrace Fuller’s internal morality are not only drawn to the fact that the principles of legality (that laws ought to be clear, prospective, obeyable and so on) specify success conditions for the performance of the law’s function of guiding human behaviour (which, in general, they assume is law’s essential function).^55^ They are also drawn to the deeper moral idea that underlies a legal system’s commitment to such principles: the idea that, by guiding human behaviour through laws that are clear, prospective, obeyable and so on, the legal system relies on and sustains the subjects’ dignity as autonomous rational agents.^56^ Jeremy Waldron talks about the agents’ capacity of ‘self-application’ (of norms) when referring to this feature of legal systems, and treats it as a necessary feature of any system of ruling worthy of being called ‘law’:

Self-application is an extraordinarily important feature of the way *legal systems* operate. They work by using, rather than short-circuiting, the agency of ordinary human individuals. They count on people’s capacities for practical understanding, for self-control, for self-monitoring and modulation of their own behavior in relation to norms that they can grasp and understand … The pervasive emphasis on self-application is … definitive of law, differentiating it sharply from systems of rule that work primarily by manipulating, terrorizing or galvanizing behavior.^57^

I would emphasise Waldron’s reference to the ‘pervasive emphasis’ on self-application. The way I understand it, this refers to the fact that properly called legal systems do not always have to rule through laws that are clear, public, obeyable and so on. Sometimes a legal system can depart from the principles of the internal morality. This flows from Fuller, who said that only ‘A total failure’ in any of his principles of legality ‘results in something that is not properly called a legal system at all’,^58^ thus leaving the threshold of legality imprecisely defined (when a failure is ‘a total failure’?). But Waldron’s point is that the legal system must display a ‘pervasive emphasis’ on this mode of ruling, so that we can differentiate properly ‘legal’ ruling from ruling which relies primarily on terror or manipulation. The normative standard is imprecise because it refers to legal systems as a whole, which are incredibly complex institutional systems, but that does not mean it is unhelpful to make a moral point or one that is entirely devoid of moral content.^59^

Despite the imprecision of this standard of legality, legal positivists who embrace it in general agree that there is a very tight connection between widespread formal defects and the systematic denial of the subjects’ agency or dignity, and that it is this last fact, more than the widespread formal defects themselves, that makes a system of ruling thus plagued with pathologies non-legal.^60^

There is a stronger way of putting this last point, by insisting that the very idea of a ‘responsible agent’ is somehow constructed or facilitated by the legal system as long as it abides by the principles of legality.^61^ Dyzenhaus appears to want to take the idea this far,^62^ as he puts forward a similar claim: that laws that fail to live up to the principles of legality put the legal subjects that are the direct addressees of those laws outside (what he calls) the ‘jural community’.

Now, this idea of the jural community does not have much content in itself, other than comprising all ‘legal subjects who have full status before the law’.^63^ The weight of the notion is placed on the contrast between subjects inside the jural community and those outside, that is, second-class citizens. Dyzenhaus has many examples at hand of historical wicked legal systems—in particular, from the Nazi regime, apartheid South Africa, occupied Palestine and antebellum United States^64^—with institutionally supported distinctions between first- and second-class citizens. These examples allow him to leave the category of a ‘full legal subject’ somewhat vague, because it is intuitively clear from the examples what he means by it.

But bringing these examples to the table does not necessarily advance Dyzenhaus’s case. Again, he wants to say that the judges of a healthy legal system have the duty to apply the principles of legality alongside positive law, implementing a Dworkinian method of legal interpretation. But clearly he wants to do more than simply declare that defective laws for formal reasons are not to be applied by judges so committed to the internal morality of law. He also wants to say that the reason behind this duty of judges is that applying formally defective laws puts certain subjects ‘outside the jural community’.

At this point, one is tempted to ask whether this is possible at all—for judges in a healthy legal system to identify laws that fall short of the standards of legality and that also, and *for this very reason*, deny some subjects the status of full legal subject of law. Considering Dyzenhaus’s examples (of cases where some individuals have been placed, by legal means, outside the jural community), it appears that some substantive element needs to be introduced for judges to arrive at the conclusion Dyzenhaus wants them to arrive at: perhaps some notion of a discriminatory intent underlying the formally defective laws in question. But this is a difficult balance to strike, because the more substantive elements one introduces into the account, the less it looks like a positivist theory and the more it starts to resemble a natural law theory.

Compare this with the approach of Jonathan Crowe, who says that individual (putative) laws can be deemed ‘defective’ as such for reasons of both form and content. The former is the case if a putative law is incomprehensible, imposes contradictory requirements or is otherwise impossible to follow. The latter case will appear depending on one’s conception of what morality covers. Crowe’s example is a law that requires all parents to immediately kill their eldest child.^65^

The problem with following Crowe with regard to the judge’s duty in the face of formally defective laws is that this approach risks losing sight of the moral reasons that make the principles of the rule of law relevant for sustaining the subjects’ dignity in the first place. Crowe’s examples are merely illustrative. Is Dyzenhaus willing to go so far as to say that every law that falls short of the principles of legality denies the subjects’ dignity or, in his preferred terms, puts the subjects outside the jural community? Does every retroactive law have this effect? Every non-public law?

My point is that the balance Dyzenhaus wants to strike might be too delicate or too difficult to be feasible. He wants judges to be mindful of formal principles of legality for substantive reasons, but he does not want to add these substantive reasons to the account. On the other hand, to treat formally defective laws as non-legal in Crowe’s sense is not expressive enough of the role Dyzenhaus believes the principles play: allowing subjects of law to stand on an equal footing with all the other members of the jural community.

Besides this issue of feasibility, Dyzenhaus’s account of the duty of judges also lacks an explanation of the causal relationship between isolated instances of application of ‘very unjust laws’ and the displacement of a group of subjects outside the jural community. He says that it is an element of the proper ‘judicial virtues’ to manifest the legal officials’ ‘fidelity to law’ (a notion popularised by Fuller) ‘by interpreting the positive law in light of’ the principles of legality.^66^ This is a sound view, but it does not establish the causal relation one could ask for.

One way to establish that causal relation and strengthen the case for the judge’s duty to apply the principles of legality goes in the form of a slippery slope argument. Let me explain. One could suggest that the duty of judges to consider the principles of legality is supported in a slippery slope style of argument in the sense that allowing for a very unjust ‘law’ to be applied in a particular case risks an otherwise healthy legal system descending into ‘wickedness’. Legal positivists committed to law’s internal morality could accept this. After all, the threshold that defines legality and non-legality is extremely vague, but there still needs to be a way of determining when a legal system moves along the continuum from legality into non-legality.

Indeed, one central feature of legal theories that define the essence of legality by resorting to ideal types of law (as is the case of the legal positivism committed to law’s internal morality) is that instances of creation (and application?) of law affect and change the overall quality of a legal system in a given time: ‘good’ elements move the system as a whole closer to the ideal type and ‘bad’ elements move it to the opposite extreme, closer to the very unjust legal system. Legality, in these theories, is a matter of degree, and moves along a continuum. Dyzenhaus appears to follow this style of reasoning. He says:

The Rule-of-Law State is … at one end of a continuum of legality. So long as a state is on that continuum, those who are subject to its law will be part of a jural community … To the extent that [the bonds of a jural community, facilitated by both positive law and principles] are lacking in a state, the closer it will move to the other end of the continuum, until the point where it falls right off …^67^

If we understand Dyzenhaus as saying that isolated instances of law-application of very unjust laws contribute to moving the healthy legal system closer to its very unjust counterpart, his case for supporting the judge’s duty to apply the principles of legality is somewhat strengthened. But note that, even if this is the case, the main problem of feasibility (which can be understood as a problem of both empirical and theoretical feasibility) remains unanswered. The issue remains: is the ‘very unjust law’ simply the formally defective law (incomprehensible, contradictory, impossible to follow, etc), or must the law display some discriminatory feature (made incomprehensible for a class of citizens?) for it to qualify as ‘very unjust’?

Clearly Dyzenhaus would want to opt for the latter. But again, adding substantive elements to the account takes it dangerously close to the natural law camp. And Dyzenhaus no longer believes natural law or moral principles to be helpful to ascertain law’s authority. This is how anti-positivists reason. Now this takes me conveniently to my next and final point.

## 5. Confronting Anti-positivists

Much of *The Long Arc of Legality* has been published previously. The first chapter in particular (where the idea of very unjust law as ‘a puzzle’ and as the most pressing problem for legal theory is introduced) is composed of previously published work, except for the final section, titled ‘Taking Law Seriously’.^68^ This section seems to have been written especially for the book. Here Dyzenhaus charges against contemporary anti-positivism—the moral impact theory of Mark Greenberg,^69^ the eliminativist approach of Scott Hershovitz^70^ and the pure legal interpretivism of Nicos Stavropoulos^71^—for wanting ‘to go beyond Dworkin to the point where … all that matters is a one-system picture of morality’.^72^ I have suggested already that for anti-positivists this is the most faithful interpretation of Dworkin’s theory as a whole. But Dyzenhaus dismisses it as an attempt, at best, to reinvent Radbruch’s version of the *lex iniusta* maxim.^73^

Furthermore, Dyzenhaus claims anti-positivists get Dworkin wrong by attempting to eliminate the distinction between ‘background rights’ and ‘legal rights’ that Dworkin, according to Dyzenhaus, wished to defend even in *Justice for Hedgehogs*.^74^ The way I understand it, he is saying that anti-positivists miss the role that can be ascribed to positive law under a Dworkin-I reading of Dworkin’s jurisprudence.

This might look like a peripheral point in Dyzenhaus’s overall proposal. But insofar as contemporary anti-positivists are thought to be reclaiming the ‘correct’ interpretation of Dworkin, which has a major role to play in Dyzenhaus’s narrative, it appears they are a worthy rival to address. The truth is that the disagreement between Dyzenhaus and contemporary anti-positivists does not really revolve around who gets Dworkin right. While it is true, as I pointed out earlier, that anti-positivists claim to elaborate their views on the law from Dworkin’s jurisprudence, they depart from it in many important aspects.^75^ Their move is to turn Dworkin’s claims about the role of principles and integrity into metaphysical claims about law’s content, a matter which is controversial even among interpreters of Dworkin.^76^

So, the disagreement goes far beyond what Dworkin, the man, really meant to say. Anti-positivists wish to deny the very starting point of legal positivism’s reflections about law: the prior existence of ‘law’ as an institutional fact with differentiating essential features and functions. In other words, they wish to deny there is something like a ‘legal ontology’.

Dyzenhaus does not seem to realise that the disagreement is metaphysical. At one point, he states that it is only the ‘moral realism which at the moment travels with pure interpretivism [which assumes that moral facts] are both universal and timeless’ that gives the theory some theoretical plausibility.^77^ But this is not necessarily what anti-positivists believe. For them, moral principles or value facts might be open to essential contestability, for this is an inescapable fact of human life. They might reply that perhaps Dyzenhaus gets the moral demands to observe law’s internal morality wrong. The issue for them is not about finding moral facts that are universal and timeless, but coming to terms with the fact that ‘law’ is whatever our morality determines it to be. For anti-positivists, ‘legal theory’ is only about determining properly ‘legal content’ by determining when subjects are faced with a legal obligation. Moral principles or value facts are the irreducible sources of normativity and thus we can only appeal to them when determining whether a legal obligation exists.

Stavropoulos would identify Dyzenhaus’s approach as a form of ‘hybrid-interpretivism’ insofar as it subjects valid legal action to further moral evaluation.^78^ But crucially, even in this hybrid form, such a theory of law takes as the starting point of legal theorising a legal ontology which specifies essential features and functions of putatively legal institutions. This fact, Stavropoulos states, impedes his anti-positivist version of interpretivism (‘pure legal interpretivism’) from even entering the discussion. He says: ‘If the orthodoxy [basically this starting ontological assumption of legal positivism] is right on [its view of why legal practice matters], interpretivism cannot get started.’^79^ That his anti-positivism ‘cannot get started’ if we assume that law has ontological-institutional existence is demonstrative of the fact that the disagreement is methodological to its very core. If someone puts forward this ontological assumption, for Stavropoulos, both sides are already talking about different things.

The way I see it, Dyzenhaus is committed, albeit implicitly, to an alternative metaphysics, one associated with the so-called ‘artifactual nature’ of law,^80^ according to which the institutional existence of law already determines its essential features and functions. Of course, anti-positivists do not deny the existence of such institutions, but they reserve the judgment to call them ‘law’ until it is verified that they actually fulfil the function of making the world morally better for its subjects.

It is important to note that this disagreement is metaphysical (and perhaps for this reason unsolvable)^81^ because, looked at from the outside (from outside legal philosophy), it appears to rest in a minor, perhaps simply terminological, difference. Dyzenhaus, like most theorists who explicitly or implicitly rely on the image of law as an artifact, acknowledges that law exists ‘out there’ (as Andrei Marmor says, ‘There is a sense in which laws clearly exist’, at least if only ‘entirely in our minds’^82^), but he also, like anti-positivists, reserves the ultimate judgment of ‘legality’ for the posterior moment where we assure ourselves that the institutional action putatively called ‘law’ passes the normative test of the internal morality. As I said, anti-positivists also acknowledge the existence of institutions and institutional action, but in order to determine conclusively whether a given action counts as ‘law’ (and not just brute coercion), we must wait and see what a judge decides in a particular case, after evaluating all prior institutional action against the background of the relevant moral principles or value facts. So, given that for both approaches every imputation of ‘legality’ is conditional upon moral evaluation, an outsider might get lost as to what both sides believe their differences to be really about.

Since the clash is metaphysical, I cannot aim to solve it here, in part because one’s metaphysical leanings on this matter are (I believe) ideologically mediated.^83^ But if there is anything to be said in favour of Dyzenhaus’s implicit metaphysics, it is that it is more ‘democratic’, in the specific sense that it allows everyone to take a first guess as to ‘where’ the law is, even if the judgment is conditional upon later verification. The anti-positivist’s metaphysics allows only the one in possession of all the knowledge of the relevant value facts and all the prior institutional acts to even take the first guess (in most accounts, this individual is always the judge, sometimes also the lawyer).

## 6. Conclusion

I believe Dyzenhaus’s *The Long Arc of Legality* convincingly shows that the best version of legal positivism is the one which includes a reference to law’s internal principles of legality. Dyzenhaus has contributed throughout his career to uncovering this aspect of legal positivism, and this book is his best attempt yet to elaborate on it. From the number of legal theorists he discusses in the book (well beyond Hobbes, Kelsen, Hart, Fuller and Dworkin), it is evident that it has been a long and excruciating journey. ‘The long arc of legality’ can be understood also as a reference to Dyzenhaus’s own journey towards perfecting his account of law.

Readers of the book will have to judge whether the project of bringing the internal morality of law into the judicial function succeeds. I have expressed some doubts here. There are also mighty legal positivist alternatives to consider, the most prominent of which is the democratic version, according to which there are moral reasons for judges to show some fidelity to the text of legislated law insofar as this law expresses the will of the subjects. This view easily combines the insights of Fuller’s formal principles of legality because it preserves them as tools to assess legal systems as a whole, thus allowing the principles that aid the judge’s interpretation of legal texts to be more substantive in nature.^84^ In Dyzenhaus’s version, the judge seems to owe direct fidelity to law’s artifactual nature, insofar as the artifact accomplishes its fundamental purpose: to rule subjects in a proper manner.

Legal positivism will continue to evolve. But whatever path it ends up taking, leaning towards either the democratic version briefly outlined or Dyzenhaus’s version, *The Long Arc of Legality* has certainly made a decisive contribution in shaping contemporary legal positivism’s identity as a theory that understands law to be an artifact whose main purpose is to guide human behaviour in a way that respects and sustains the subjects’ dignity.

This reconstruction might make legal positivism a more attractive theory than anti-positivism, but, as I said, that judgment will depend in the end on one’s metaphysical (that is, ideological) leanings. However, this theoretical debate, still prominent in specialised academic circles, should not distract us from the underlying political aim that actually brings all these contemporary legal theories together. Contemporary legal positivists and anti-positivists alike agree that the identification of proper ‘law’ is, in an important sense, intertwined with the legitimation of coercive institutional action. Both theories agree that ‘law’ has to be constructed as something more than coercive institutional action, and its actions must be morally justifiable to each person. In other words, both share the same political-liberal mindset. Anti-positivists are not simply offering a metaphysically extravagant theory. Their ultimate goal is to present ‘law’ as something that must improve the moral situation of subjects, and disavow coercion when coercion is not morally justifiable.

Politically speaking, Dyzenhaus shares the anti-positivist’s overall normative aim. So, under a conciliatory reading, what we get from this debate is that contemporary legal theory has not lost sight of the fact that a philosophy of law (the inquiry to understand what ‘law’ is) always entails a political philosophy about the legitimacy of state action.

## Footnotes

1
*Legality* 25.

2 ibid.

3 D Dyzenhaus, ‘Dworkin and Unjust Law’ in W Waluchow and S Sciaraffa (eds), *The Legacy of Ronald Dworkin* (OUP 2016) 136.

4 A Marmor, ‘What’s Left of General Jurisprudence? On Law’s Ontology and Content’ (2019) 10 Jurisprudence 151.

5
*Legality* 1.

6 ibid.

7 Marmor, ‘What’s Left of General Jurisprudence?’ (n 4) 153.

8 K Ehrenberg, *The Functions of Law* (OUP 2016) 188 (citing J Raz, ‘The Problem of Authority: Revisiting the Service Conception’ (2006) 90 Minn L Rev 1003, 1018).

9
*Legality* 159.

10
*Legality* 1.

11
*Legality* 2.

12
*Legality* 2 and 297.

13 L Fuller, *The Morality of Law*, revised edn (Yale UP 1969) 46–91. See *Legality* 18.

14
*Legality* 4.

15
*Legality* 3.

16 One can say that what Dyzenhaus finds missing in Raz’s account of legal authority is the public nature of legal institutions and of legal action. His reservations are echoed in the literature that criticises Raz’s ‘service conception of authority’ and the ‘normal justification thesis’ precisely because these theses dispense with the institutional existence of law (as a prior condition of its authority). For this specific critique, see A Marmor, ‘The Dilemma of Authority’ (2011) 2 Jurisprudence 129. Similarly, J Waldron, ‘Authority for Officials’ in L Meyer, S Paulson and T Pogge (eds), *Rights, Culture and the Law: Themes from the Legal and Political Philosophy of Joseph Raz* (OUP 2003) 61–6.

17
*Legality* 161–79.

18
*Legality* 149–61 and 186–223.

19
*Legality* 223–96.

20
*Legality* 23.

21 In what follows, I speak of ‘judges’ and the ‘judicial function’ for simplicity, but this should be understood as referring to any official in charge of applying law.

22
*Legality* 41.

23 M Murphy, *Natural Law in Jurisprudence and Politics* (CUP 2006) 8–24.

24
*Legality* 3–4.

25 Dyzenhaus is much committed to the idea that law’s authority is grounded in a social contract between those who hold institutional power and the subjects (*Legality* 179–86). This is why he devotes ch 2 of the book to vindicating Hobbes’s political philosophy of the state (*Legality* 88–148).

26 Throughout the book, Dyzenhaus claims to be following a pragmatist approach to law. He offers pragmatist interpretations of Fuller and Hart, although he applies the label more explicitly to his reading of Kelsen (*Legality* 27–9). Dyzenhaus claims that his pragmatist approach is in the service of a particular interpretation of Kelsen’s idea that legal action has to be theoretically reconstructed as political action serving some purpose, thus constructing an analogy between Hobbes’s social contract and Kelsen’s ‘basic norm’ (*Legality* 283). He also calls this approach ‘dynamic’ (as opposed to ‘static’), which means it integrates the factual and the normative sides of law (*Legality* 22–3). Thomas Bustamante’s review of *The Long Arc of Legality* highlights this methodological aspect of the book: T Bustamante, ‘Interpretive Authority and the Kelsenian Quest of Legality’ *Jotwell* (5 July 2022).

27
*Legality* 319 and 378.

28 HLA Hart, *The Concept of Law* (2nd edn, Clarendon Press 1994).

29
*Legality* 68–9.

30 Adam Tucker, commenting on Dyzenhaus’s views on legal positivism, says that ‘Dyzenhaus is the standard bearer of a school of thought that associates the philosophy of legal positivism with undesirable consequences in real life politics’: A Tucker, ‘The Politics of Legal Positivism: A Reply to David Dyzenhaus (2013) 38 Australian Journal of Legal Philosophy 74.

31 D Dyzenhaus, *Hard Cases in Wicked Legal Systems: South African Law in the Perspective of Legal Philosophy* (Clarendon Press 1991).

32 D Dyzenhaus, ‘Why Positivism is Authoritarian’ (1992) 37 Am J Juris 83.

33 ibid 105.

34 ibid 111. Emphasis in the original.

35 D Dyzenhaus, ‘The Legitimacy of Law: A Response to Critics’ (1994) 7 Ratio Juris 80, 90.

36 J Dickson, ‘Is Bad Law Still Law? Is Bad Law Really Law?’ in M Del Mar and Z Bankowski (eds), *Law as Institutional Normative Order* (Ashgate 2009) 163.

37 J Dickson, *Elucidating Law* (OUP 2022) 102–3.

38 ibid 85–90.

39 ibid 90–4 and 122–33.

40 ibid 94.

41 ibid 83.

42 ibid 98.

43 In Trevor Allan’s terms, the problem with the argument of Dworkin’s *Law’s Empire*, which the fact of ‘wicked laws’ makes evident, is a contradiction between Dworkin’s theory of the grounds of law and his theory of law’s force: TRS Allan, ‘Law, Justice and Integrity: The Paradox of Wicked Laws’ (2009) 29 OJLS 705; R Dworkin, *Law’s Empire* (Harvard UP 1986).

44 R Dworkin, *Justice for Hedgehogs* (Harvard UP 2011).

45 Dyzenhaus, ‘Dworkin and Unjust Law’ (n 3) 153.

46 ibid 153–7.

47 Dworkin, *Law’s Empire* (n 43).

48 To cite just a couple of examples of prominent legal positivists: J Waldron, ‘Legislating with Integrity’ (2003) 72 Fordham L Rev 373, 376 (stating that the gravitational force of Dworkinian legal principles ‘bears on questions of rule-interpretation or hard cases where we appear to be torn between the effect of different legal provisions’); J Gardner, ‘Law’s Aims in *Law’s Empire*’ in S Hershovitz (ed), *Exploring Law’s Empire: The Jurisprudence of Ronald Dworkin* (OUP 2008) 207–23 (arguing that what Dworkin offers in *Law’s Empire* is a theory of constructive interpretation which is entirely compatible with legal positivism, and concluding in a provocative note that legal philosophers ‘should be pleased to welcome Dworkin back into the best tradition of thinking about law, which is the legal positivist tradition’).

49 R Dworkin, ‘The Model of Rules’ (1967) 35 U Chi L Rev 14.

50 R Dworkin, ‘Social Rules and Legal Theory’ (1972) 81 Yale LJ 855. Reprinted as ‘The Model of Rules II’ in R Dworkin, *Taking Rights Seriously* (Harvard UP 1978).

51 N Stavropoulos, ‘Why Principles?’ (2007) Oxford Faculty of Law Legal Studies Research Paper Series No 28; N Stavropoulos, ‘The Debate That Never Was’ (2017) 130 Harv L Rev 2088.

52 L Fuller, *The Law in Quest of Itself* (The Foundation Press 1940) 137–8.

53 Fuller, *The Morality of Law* (n 13) 82.

54 K Rundle, *Forms Liberate: Reclaiming the Jurisprudence of Lon L Fuller* (Hart Publishing 2013) 81 (emphasis added).

55 See eg A Marmor, *Law in the Age of Pluralism* (OUP 2007) ch 1.

56 The first one to see this, in relation to Fuller, was Joseph Raz, in his influential 1977 essay ‘The Rule of Law and Its Virtue’ reprinted in his *The Authority of Law* (OUP 2009) 210–29. Here, Raz, probably echoing Fuller’s statement that ‘Every departure from the principles of the law’s inner morality is an affront to man’s dignity as a responsible agent’ (Fuller, *The Morality of Law* (n 13) 162), said that ‘observance of the rule of law is necessary if the law is to respect human dignity’, which entails ‘treating humans as persons capable of planning and plotting their future’. Others followed later in similar lines. See eg D Luban, ‘The Rule of Law and Human Dignity: Re-examining Fuller’s Canons’ (2010) 2 HJRL 29–47; J Waldron*, Dignity, Rank, and Rights* (OUP 2012); B Celano, ‘Law as Power: Two Rule of Law Requirements’ in W Waluchow and S Sciaraffa (eds), *Philosophical Foundations of the Nature of Law* (OUP 2013) 129–51.

57 Waldron (n 56) 52 (emphasis added).

58 Fuller, *The Morality of Law* (n 13) 39.

59 I think it is right to understand Fuller’s idea of the rule of law as Nigel Simmonds does: as expressing both a baseline of legality (the minimum content of a proper legal system) and a moral aspiration (for the legal system to improve as far as possible): N Simmonds, *Law as a Moral Idea* (OUP 2007) 65. Jeremy Waldron makes a similar point, stating that even when a system of ruling is ‘recognizable as a legal system’ insofar as it abides by the minimum requirements of the rule of law, ‘we may still demand more from that system’, adding further that ‘The fact that we work with a roughly defined threshold for a system of governance to count as law does not mean that we rest satisfied with these minimum credible achievements. There is always room for improvement’: J Waldron, ‘The Concept and the Rule of Law’ (2008) 43 Ga L Rev 1, 45.

60 Dan Priel has promisingly attempted to establish an empirical connection between respect for Fuller’s principles of legality, respect for subjects’ personhood and the likelihood of a legal system descending into tyranny: D Priel, ‘Lon Fuller’s Political Jurisprudence of Freedom’ (2014) 10 Jerusalem Review of Legal Studies 18, 37–9.

61 See K Rundle, ‘Legal Subjects as Juridical Persons: Developing Public Legal Theory through Fuller and Arendt’ (2014) 43 Netherlands Journal of Legal Philosophy 212, 222.

62
*Legality* 300.

63
*Legality* 30.

64
*Legality* 322–46.

65 J Crowe, ‘Defects and Failures in Legal Artifacts’ in L Burazin and others (eds), *The Artifactual Nature of Law* (Edward Elgar 2022) 45.

66
*Legality* 378.

67
*Legality* 323.

68
*Legality* 79–87.

69 M Greenberg, ‘The Moral Impact Theory of Law’ (2014) 123 Yale LJ 1288.

70 S Hershovitz, ‘The End of Jurisprudence’ (2015) 124 Yale LJ 1160.

71 N Stavropoulos, ‘Legal Interpretivism’, *The Stanford Encyclopedia of Philosophy* (2021).

72
*Legality* 80.

73
*Legality* 83.

74
*Legality* 86–7.

75 Stavropoulos acknowledges that his understanding of the role of principles ‘differs from the one developed by Dworkin’, although he thinks that his conception ‘remains faithful to the spirit of his arguments’: Stavropoulos, ‘Why Principles?’ (n 51) 2. Greenberg also states that his theory departs from Dworkin’s in fundamental aspects.

76 For a ‘Dworkinian’ critique to Mark Greenberg’s anti-positivist theory, see T Bustamante, ‘Law, Moral Facts and Interpretation: A Dworkinian Response to Mark Greenberg’s Moral Impact Theory of Law’ (2019) 32 Canadian Journal of Law & Jurisprudence 5. Bustamante is categorial: ‘Dworkin’s interpretive theory of law is not and should not be transformed into a metaphysical theory of law’ (17).

77
*Legality* 87.

78 Stavropoulos, ‘Legal Interpretivism’ (n 71) (under ‘Hybrid Interpretivism’).

79 N Stavropoulos, ‘Obligations, Interpretivism, and the Legal Point of View’ in Andrei Marmor (ed), *The Routledge Companion to Philosophy of Law* (Routledge 2012) 77.

80 L Burazin, K Himma and C Roversi (eds), *Law as an Artifact* (OUP 2018).

81 It doesn’t need to be necessarily unsolvable. Benjamin Ruiz García is currently working on a PhD dissertation aimed at demonstrating that legal anti-positivism (mainly Greenberg’s version) is compatible with and actually presupposes the artifactual nature of law. I thank Benjamin for sharing his unpublished manuscript ‘The Moral Impact Theory and the Notion of Law as an Artifact’.

82 A Marmor, ‘Law, Fiction, and Reality’ in Burazin, Himma and Roversi (n 80) 44.

83 I follow Dan Priel’s observation that ‘the claim that law is an artifact is an ideological claim’, by which he means that it is ‘not a universal truth about law wherever and whenever we find it, but a normative stance’: D Priel, ‘Not All Law Is an Artifact. Jurisprudence Meets the Common Law’ in Burazin, Himma and Roversi (n 80) 240. I expand this to the rival metaphysical claim of anti-positivists regarding the nature of law, ie that ‘law’ is the relevant institutional action assessed through the lens of the relevant value facts. For reasons of space, I cannot argue for this view in detail, but it seems right to think that opting for one metaphysical view of law rather than the other (assuming that both views explain the phenomenon in plausible ways) is best described as taking a normative stance. In that (still obscure) sense, opting for one metaphysical view is a matter of ideology.

84 This is essentially Waldron’s position. See J Waldron, ‘Can There Be a Democratic Jurisprudence?’ (2009) 58 Emory LJ 675.

## Notes

The author wishes to thank David Dyzenhaus and Andreas Vassiliou for comments to a previous draft.

